# Aquaporin-3 potentiates allergic airway inflammation in ovalbumin-induced murine asthma

**DOI:** 10.1038/srep25781

**Published:** 2016-05-11

**Authors:** Kohei Ikezoe, Toru Oga, Tetsuya Honda, Mariko Hara-Chikuma, Xiaojun Ma, Tatsuaki Tsuruyama, Kazuko Uno, Jun-ichi Fuchikami, Kiminobu Tanizawa, Tomohiro Handa, Yoshio Taguchi, Alan S. Verkman, Shuh Narumiya, Michiaki Mishima, Kazuo Chin

**Affiliations:** 1 Department of Respiratory Medicine, Kyoto University Graduate School of Medicine, Sakyo-ku, Kyoto 606-8507, Japan; 2 Department of Respiratory Care and Sleep Control Medicine, Kyoto University Graduate School of Medicine, Sakyo-ku, Kyoto 606-8507, Japan; 3 Department of Dermatology, Kyoto University Graduate School of Medicine, Sakyo-ku, Kyoto 606-8507, Japan; 4 Center for Innovation in Immunoregulative Technology and Therapeutics (AK project), Kyoto University Graduate School of Medicine, Sakyo-ku, Kyoto 606-8501, Japan; 5 Core Research for Evolutional Science and Technology (CREST) Laboratory, Medical Innovation Center, Kyoto University Graduate School of Medicine, Sakyo-ku, Kyoto 606-8501, Japan; 6 Center for anatomical, forensic and pathology research, Kyoto University Hospital, Sakyo-ku, Kyoto 606-8501, Japan; 7 Louis Pasteur Center for Medical Research, Sakyo-ku, Kyoto 606-8225, Japan; 8 Bioresearch Center, CMIC Pharma Science Co., Ltd., Hokuto-shi, Yamanashi 408-0044, Japan; 9 Department of Respiratory Medicine, Tenri Hospital, Tenri-shi, Nara 632-8552, Japan; 10 Departments of Medicine and Physiology, University of California, San Francisco, CA 94143, USA

## Abstract

Oxidative stress plays a pivotal role in the pathogenesis of asthma. Aquaporin-3 (AQP3) is a small transmembrane water/glycerol channel that may facilitate the membrane uptake of hydrogen peroxide (H_2_O_2_). Here we report that AQP3 potentiates ovalbumin (OVA)-induced murine asthma by mediating both chemokine production from alveolar macrophages and T cell trafficking. AQP3 deficient (AQP3^−/−^) mice exhibited significantly reduced airway inflammation compared to wild-type mice. Adoptive transfer experiments showed reduced airway eosinophilic inflammation in mice receiving OVA-sensitized splenocytes from AQP3^−/−^ mice compared with wild-type mice after OVA challenge, consistently with fewer CD4^+^ T cells from AQP3^−/−^ mice migrating to the lung than from wild-type mice. Additionally, *in vivo* and *vitro* experiments indicated that AQP3 induced the production of some chemokines such as CCL24 and CCL22 through regulating the amount of cellular H_2_O_2_ in M2 polarized alveolar macrophages. These results imply a critical role of AQP3 in asthma, and AQP3 may be a novel therapeutic target.

Asthma is characterized by chronic inflammation of the airways where many cells and cellular elements are involved, and is associated with increased airway hyperresponsiveness (AHR). Although eosinophilic inflammation is a characteristic feature of asthma, T cells recruited into the airways orchestrate the inflammatory reaction through their secretion of cytokines and other mediators^1^. T helper type 2 (Th2) cytokines, such as interleukin (IL)-4 and IL-13, are involved in the class-switching of B cells to immunoglobulin E (IgE) synthesis, recruitment of mast cells, and maturation of eosinophils and basophils^2^. IL-4 and IL-13 can also induce the polarization of macrophages to alternatively activated macrophages (M2 macrophages). M2 macrophages are important in the attraction of cells to inflammatory foci, suppression of Th1 responses, sampling of the microenvironment by endocytosis, and orchestration of tissue repair^3^.

Oxidative stress plays a pivotal role in the pathogenesis of asthma. Reactive oxygen species (ROS), including hydrogen peroxide (H_2_O_2_), may initiate and augment airway inflammation^4^. ROS increase airway smooth muscle contraction and stimulate mucin secretion. Many cell types, including lymphocytes and macrophages, are involved in the increased production of ROS in asthma. Recently, oxidative stress was suggested to be an important factor in the development of corticosteroid insensitivity, in relation to severe asthma^5^.

Aquaporins (AQPs) are small integral membrane proteins that transport water across cell plasma membranes^6^. Of these, aquaglyceroporins, including aquaporin-3 (AQP3), also transport small uncharged molecules such as glycerol. AQP3 is localized in various tissues, including the kidney, skin, gastrointestinal tract, and respiratory tract^7^. As to its physiological roles, AQP3 is known to be essential for the urinary-concentrating mechanism in the kidney, and AQP3-mediated glycerol transport is important for skin hydration^8^,^9^. Recently, AQP3 was found to facilitate the membrane uptake of H_2_O_2_, and influence the downstream cell signaling cascade in mammalian cells^10^. We also have reported that AQP3-mediated H_2_O_2_ uptake is essential for chemokine-dependent T cell migration^11^.

AQP3 expression was shown to be upregulated in some murine asthma models^12^,^13^, although its role remains unknown. We hypothesized that AQP3 would contribute to the pathogenesis of asthma by regulating the amount of cellular H_2_O_2_. We tested this hypothesis using AQP3 deficient (AQP3^−/−^) mice in an ovalbumin (OVA)-induced murine asthma model. Further, we then determined that AQP3 facilitated murine asthma through mediating chemokine production from alveolar macrophages (AMs) as well as regulating T cell trafficking.

## Results

### Reduced OVA-induced allergic asthma in AQP3 deficient mice

**Figure 1:**
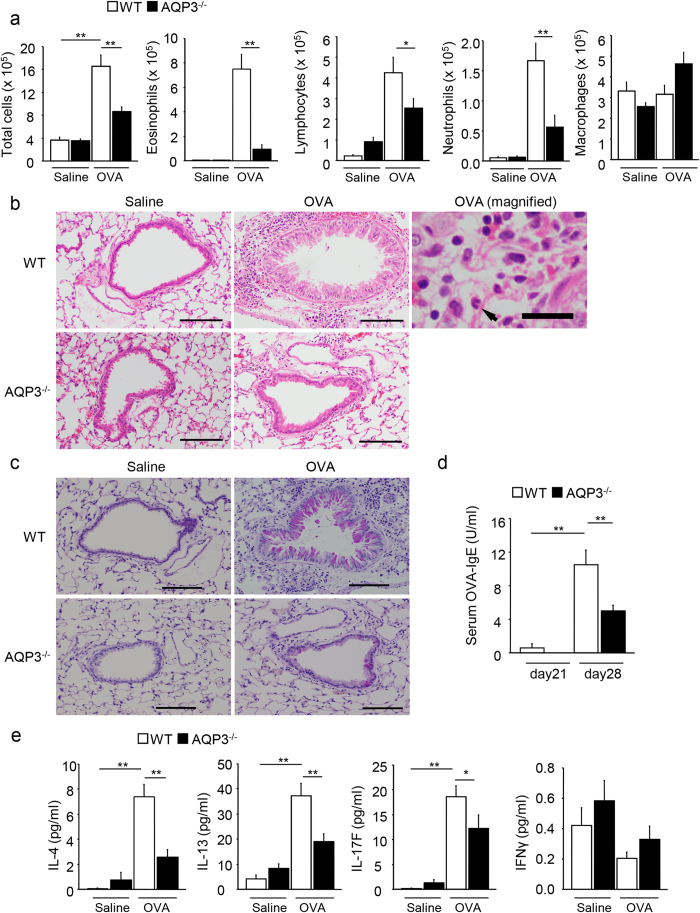
Allergic airway responses are suppressed in AQP3^−/−^ mice. **(a)** Cell population analyses in BALF by differential cell counts from WT and AQP3^−/−^ mice (n = 5 for each control group and n = 8 for each OVA-challenged group) on day 28. **(b**,**c)** Lung histology. Lungs of WT (n = 5 for each group) and AQP3^−/−^ (n = 4 for each group) mice were dissected on day 28 and stained with hematoxylin and eosin (**b**) or periodic acid-Schiff (**c**). Bars, 50 μm. The magnified figure shows the cells in the peribronchial lesion in OVA-challenged WT mice. Arrow indicates eosinophil. Bars of the magnified figure, 10 μm. **(d)** Serum levels of OVA-specific IgE. Serum concentrations of OVA-specific IgE were measured in WT (n = 5 for each group) and AQP3^−/−^ (n = 5 for each group) mice on day 21 and day 28. **(e)** Measurements of concentrations of IL-4, IL-13, IL-17F, and IFN-γ in BALF collected on day 28 (n = 5 for each control group; n = 8 for each OVA-challenged group). **P < 0.01, *P < 0.05.

After following the procedure for sensitization and challenge shown in Supplementary Fig. S1a, we compared OVA-induced allergic asthma between AQP3^−/−^ mice and wild-type (WT) mice by assessing airway inflammation using cell counts and lung sections, by evaluating airway responsiveness to methacholine, and by measuring concentrations of IgE and Th2 cytokines. The number of total cells as well as the numbers of eosinophils, lymphocytes, and neutrophils in bronchoalveolar lavage fluid (BALF) increased after OVA challenge in WT mice (Fig. 1a). However, these numbers were significantly reduced in AQP3^−/−^ mice compared with WT mice. Especially, a substantial reduction in eosinophilia, characteristic in allergic inflammation, was observed in BALF of AQP3^−/−^ mice. These results were confirmed using littermate WT control mice. The numbers of T cells, B cells, CD4^+^ T cells, and CD8^+^ T cells in BALF from OVA-challenged AQP3^−/−^ mice were also fewer than those from OVA-challenged WT mice (Supplementary Fig. S2a). Histological examination showed infiltration of inflammatory cells in the perivascular and peribronchial lesions (Fig. 1b) and mucus hypersecretion in airway epithelium (Fig. 1c) in OVA-challenged WT mice. In contrast, there was less cellularity and hypersecretion in AQP3^−/−^ mice after OVA challenge. Specific airway resistance (sRaw) to methacholine was elevated in OVA-challenged WT mice compared to non-OVA-challenged WT mice, but the elevation was significantly lower in OVA-challenged AQP3^−/−^ mice (Supplementary Fig. S2b). However, this should be interpreted with caution, as the response to methacholine in non-OVA-challenged AQP3^−/−^ mice was so flat that OVA-challenged AQP3^−/−^ mice might have had a flatter airway response due to a baseline defect not to an OVA response effect.

We measured concentrations of OVA-specific IgE in the serum and several cytokines in BALF in WT and AQP3^−/−^ mice and compared the results. Serum OVA-specific IgE levels were higher in WT mice after OVA inhalation than before OVA inhalation, while those values in AQP3^−/−^ mice also increased but remained lower than in the WT mice (Fig. 1d). In both AQP3^−/−^ and WT groups, concentrations of Th2 cytokines, IL-4 and IL-13, and the Th17 cytokine, IL-17F, in BALF increased after OVA challenge (Fig. 1e). However, OVA-challenged AQP3^−/−^ mice had lower concentrations than did WT mice, while the levels of interferon-γ, the hallmark Th1 cytokine, in BALF were comparable between WT and AQP3^−/−^ mice. Thus, the loss of AQP3 resulted in reduced OVA-induced asthma responses, resulting in less eosinophilic inflammation and decreased Th2 cytokine production.

### Impaired CD4^+^ T cell trafficking in OVA-challenged AQP3 deficient mice

**Figure 2:**
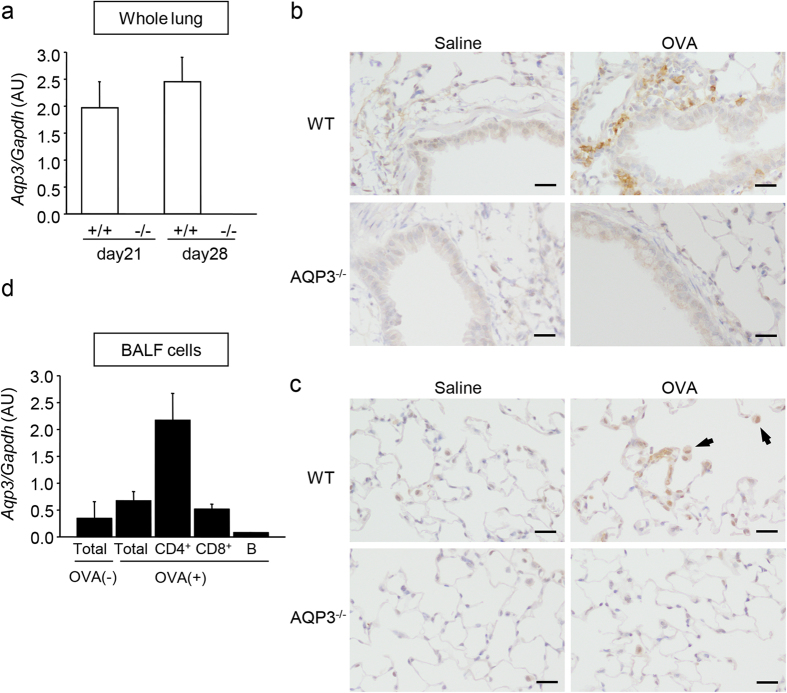
AQP3 is expressed on T cells, AMs, and epithelial cells. **(a)** mRNA expression levels of *Aqp3* in whole lungs. Total RNA was extracted from lungs obtained from mice on day 21 (n = 3 for each group) and on day 28 (n = 4 for each group). *Aqp3* mRNA was assessed by real-time RT-PCR. **(b**,**c)** Immunohistochemistry of AQP3 in the lung. Lungs of saline-challenged (n = 3) and OVA-challenged (n = 4–5) WT or AQP3^−/−^mice were dissected and stained with AQP3 antibody. Small airways (**b**) and alveolar spaces (**c**) are shown. Arrows indicate AMs. Bars, 10 μm. **(d)** mRNA expression levels of *Aqp3* in total inflammatory cells and sorted CD4^+^, CD8^+^, and B cells in BALF collected from OVA-challenged WT mice (n = 3 for each group).

The above results suggest a possibility that AQP3 is involved in allergic responses in the lung. Consistently, *Aqp3* messenger RNA (mRNA) was detected in the lungs of WT mice by real-time reverse transcription (RT)-PCR analysis, and it was considered that OVA challenge to the airway might enhance its levels (Fig. 2a). Immunohistochemistry revealed that AQP3 was expressed strongly in some inflammatory cells and weakly in airway epithelial cells in peribronchovascular lesions (Fig. 2b), and in AMs in alveolar lesions (Fig. 2c). As T cells, especially CD4^+^ T cells, play a critical role in the pathogenesis of asthma, we focused on AQP3 expression in those cells. We sorted BALF cells in OVA-challenged WT mice into CD4^+^ T cells, CD8^+^ T cells, and B cells by Auto MACS and examined *Aqp3* mRNA levels in these cells by real-time RT-PCR. Of these, CD4^+^ T cells most highly expressed *Aqp3* mRNA and B cells least expressed it after OVA challenge (Fig. 2d).

**Figure 3:**
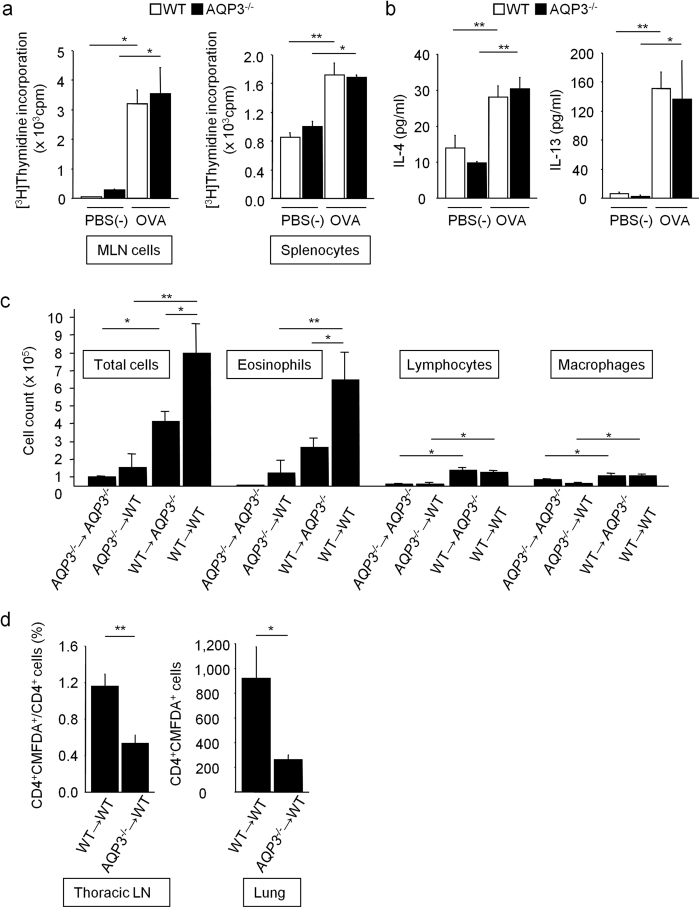
Impaired asthmatic airway inflammation is partly caused by decreased T cell trafficking in AQP3^−/−^ mice. **(a**,**b)** Splenocytes and mesenteric lymph nodes (MLN) cells from OVA-sensitized WT and AQP3^−/−^ mice were isolated on day 21 and were cultured in the presence/absence of OVA. **(a)** OVA-specific T cell proliferation assay determined by incorporation of 0.5 μCi/ml [^3^H]-thymidine for 24 h. **(b)** Levels of IL-4 and IL-13 in the supernatants of splenocytes (n = 3–4 for each group). **(c)** Adoptive transfer experiments by intravenous injection. Splenocytes from OVA-sensitized donor WT and AQP3^−/−^ mice on day 21 were injected intravenously (1 × 10^7^ cells/head) to naïve recipient WT and AQP3^−/−^ mice, then the recipient mice were challenged with OVA for 7 days. Numbers of total cells, eosinophils, lymphocytes and macrophages in BALF were determined (n = 4 for each group). **(d)** Adoptive transfer experiments to track transferred CD4^+^ T cells. Splenocytes from OVA-sensitized donor WT and AQP3^−/−^ mice on day 21 were stained with CMFDA and injected into recipient WT mice intravenously (2 × 10^7^ cells/head). After OVA challenge of these recipient mice, CD4^+^ and CMFDA^+^ cells in the thoracic lymph nodes and lungs were analyzed by flow cytometry (n = 5 for each group). **P < 0.01, *P < 0.05.

We then examined T cell proliferation and activation during the sensitization phase. Splenocytes and mesenteric lymph node cells from OVA-sensitized WT and AQP3^−/−^ mice were isolated on day 21 before the OVA challenge and were cultured in the presence and absence of OVA. The OVA-specific T cell proliferation and IL-4/IL-13 production were comparable between WT and AQP3^−/−^ mice (Fig. 3a,b). These findings suggest that AQP3 is not required for OVA-specific T cell proliferation and activation.

Next, we examined the functions of T cells during the elicitation phase by using intravenous adoptive transfer (Supplementary Fig. S1b). As shown in Fig. 3c, the transfer of OVA-sensitized AQP3^−/−^ splenocytes resulted in reduced total inflammatory cells and eosinophils in BALF in the recipient WT and AQP3^−/−^ mice compared with the results from the transfer of WT donor cells after OVA challenge. However, even though the same genotype-derived splenocytes were transferred, the numbers of total inflammatory cells and eosinophils in BALF were fewer in AQP3^−/−^ recipient mice than in WT mice after OVA challenge. Furthermore, to assess the role of AQP3 in T cell trafficking *in vivo*, we adoptively transferred the sensitized WT or AQP3^−/−^ splenocytes that were labeled with the cell-tracker dye 5-chloromethylfluorescein diacetate (CMFDA) (Supplementary Fig. S1c). Fewer CD4^+^CMFDA^+^ cells from AQP3^−/−^ mice were observed in both thoracic lymph nodes and lungs than from WT mice (Fig. 3d). These results provide evidence that AQP3 expression plays an important role in airway inflammation through its effect on CD4^+^ T cell trafficking during OVA challenge to the airway, but that is only a partial effect and other functions of AQP3 were indicated.

### Impaired chemokine production by AMs in OVA-challenged AQP3-deficient mice

**Figure 4:**
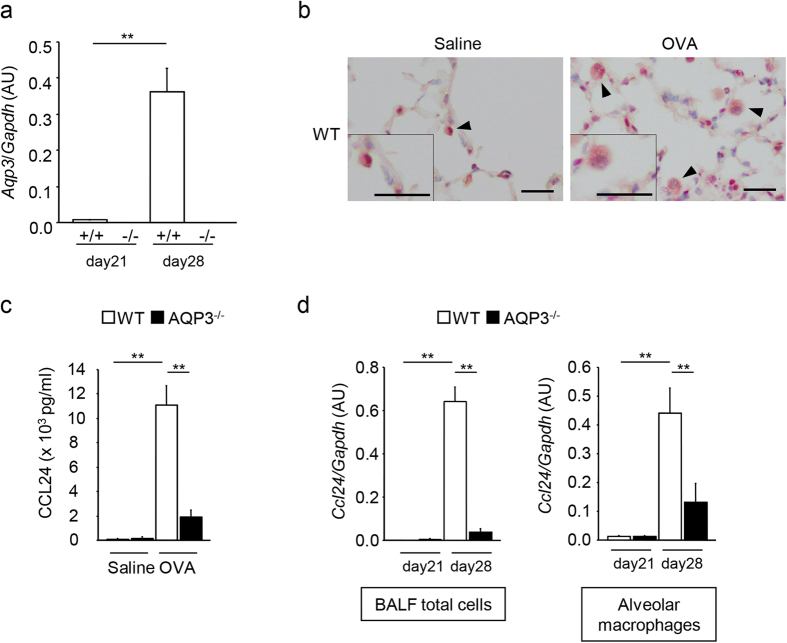
The production of CCL24 is impaired in AMs from AQP3^−/−^ mice. **(a)** *Aqp3* mRNA expression levels in AMs. mRNA expression levels of *Aqp3* in FACS-Aria sorted AMs (F4/80^+^CD11c^+^ cells) in the lungs (n = 4 for each control group; n = 6 for each OVA-challenged group). **(b)** Double staining of immunohistochemistry of AQP3 and F4/80 in the lung. Lungs of saline-challenged WT mice (left) and OVA-challenged WT mice (right) were dissected and stained with AQP3 antibody and F4/80 antibody. Arrowheads indicate AMs. Bars, 10 μm. **(c)** Concentrations of CCL24 in BALF on day 28 (n = 5 for each control group; n = 8 for each OVA-challenged group). **(d)** *Ccl24* mRNA expression levels in BALF total cells (n = 4 on day 21; n = 7 on day 28) and AMs (n = 4 on day 21; n = 6 on day 28). **P < 0.01.

We next focused on the possible contributions of AMs to allergic reactions through AQP3 expression since immunohistochemistry revealed that AQP3 was expressed in AMs (Fig. 2c). First, we examined the expression of *Aqp3* mRNA in FACS Aria-sorted AMs (F4/80^+^CD11c^+^ cells) from OVA-challenged mice by real-time RT-PCR. OVA challenge enhanced *Aqp3* mRNA expression in AMs from WT mice (Fig. 4a). We also confirmed that AQP3 was expressed in AMs (F4/80^+^ cells) in the lungs of OVA-challenged WT mice for the double staining of immunohistochemistry (Fig. 4b). Previous studies showed that AM was a major source of allergen-induced CCL24 (eotaxin-2), which is a chemoattractant for eosinophils, in mice^14^,^15^,^16^,^17^. Therefore, we measured the levels of CCL24 in BALF from WT and AQP3^−/−^ mice by ELISA and found that the levels of CCL24 in BALF significantly increased after OVA challenge in WT mice but not in AQP3^−/−^ mice (Fig. 4c). Moreover, OVA challenge also strongly enhanced *Ccl24* mRNA expression in total BALF cells and AMs in WT mice, but to significantly lesser levels in AQP3^−/−^ mice (Fig. 4d). These findings suggest that CCL24 production by AMs was impaired in OVA-challenged AQP3^−/−^ mice.

**Figure 5:**
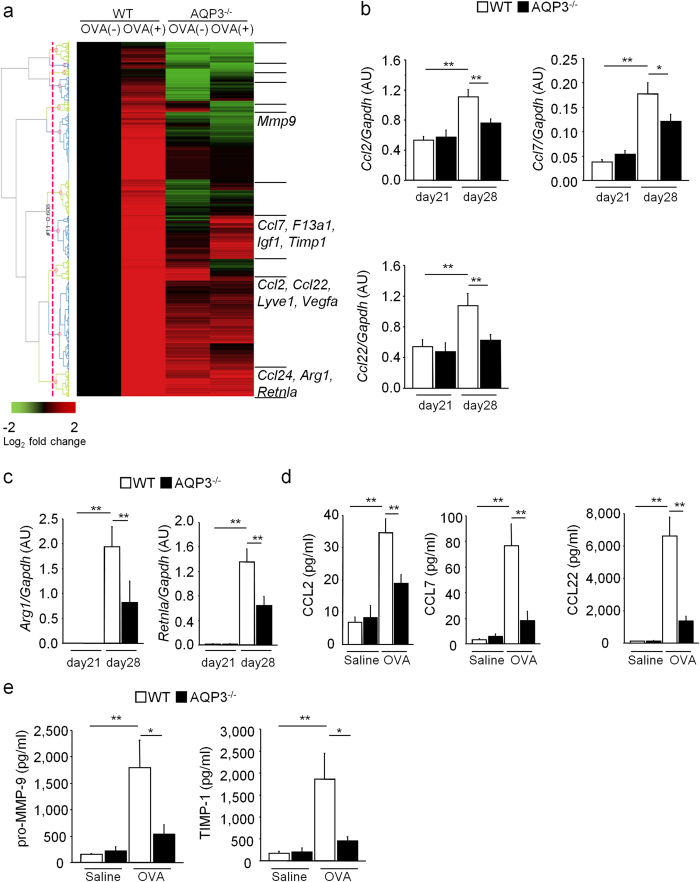
The expressions of genes involved in chemokine, M2 polarization, and tissue remodeling are decreased in AMs from OVA-challenged AQP3^−/−^ mice. (**a**) Heat map of genes (clusters 9 and 11) whose expressions were elevated after OVA-challenge in WT AMs, but not AQP3^−/−^ AMs. Genes involved in chemokine, tissue remodeling, and M2 polarization are arbitrarily shown. (**b**) mRNA expression levels of *Ccl2, Ccl7*, and *Ccl22* in AMs from OVA-challenged WT and AQP3^−/−^ mice (n = 4 on day 21; n = 6 on day 28). (**c**) mRNA expression levels of M2 marker (*Arg1* and *Retnla*) in AMs from OVA-challenged WT and AQP3^−/−^ mice (n = 4 on day 21; n = 6 on day 28). (**d**) Concentrations of chemokines (CCL2, CCL7, and CCL22) in BALF after OVA challenge in WT and AQP3^−/−^ mice (n = 5 for each control group; n = 8 for each OVA-challenged group). (**e**) Concentration of pro-MMP-9 and TIMP-1 in BALF after OVA challenge in WT and AQP3^−/−^ mice (n = 4 for each control group; n = 7 for each OVA-challenged group). **P < 0.01, *P < 0.05.

To determine the role of AQP3 in functions of AMs during OVA-induced asthma, using microarray analysis we compared the gene expression profiles of AMs in WT and AQP3^−/−^ mice before and after OVA challenge. We extracted and analyzed 8,458 genes, which we divided into 11 clusters on the basis of their induction pattern (Supplementary Fig. S3a). We focused on clusters 9 and 11 where the expression of the genes was enhanced after OVA challenge in WT AMs but not in AQP3^−/−^ AMs. We performed hierarchical clustering using the genes on clusters 9 and 11 (2,591 genes) (Fig. 5a). It was revealed that the expressions of M2 macrophage-related genes such as *Arg1* and *Retnla* (also known as *Fizz1*) were highly increased in OVA-challenged WT AMs but repressed in OVA-challenged AQP3^−/−^ AMs. Similarly, the expressions of genes related to chemokines, such as *Ccl2, Ccl7, Ccl22*, and genes related to tissue remodeling, such as *Igf1, Vegfa, Lyve1, F13a1, Mmp9*, and *Timp1*, were also highly reduced in OVA-challenged AQP3^−/−^ AMs compared to OVA-challenged WT AMs. The differential expressions of several genes were confirmed by quantitative real-time RT-PCR (Fig. 5b,c, and Supplementary Fig. S3b). In addition, we then measured the concentrations of those chemokines in BALF during OVA-induced airway inflammation. In accordance with microarray and real-time RT-PCR data for AMs, their levels were highly increased after OVA challenge in WT mice but were significantly suppressed in OVA-challenged AQP3^−/−^ mice (Fig. 5d). Levels of pro-matrix metalloproteinase-9 (MMP-9) and tissue inhibitor of metalloproteinase-1 (TIMP-1) in BALF were also increased after OVA challenge in WT mice but less increased in AQP3^−/−^ mice (Fig. 5e).

Moreover, we compared the expression of overall chemokine-related genes between groups again. Besides the chemokines described above, several chemokines were expressed highly in OVA-challenged WT AMs compared to WT AMs before OVA-challenge, and their expressions were strikingly suppressed in OVA-challenged AQP3^−/−^ AMs (Table 1). We measured the levels of some of these chemokines (CCL4, CCL11, CCL17, and CXCL1) in BALF, and confirmed that they were lower in OVA-challenged AQP3^−/−^ mice compared to OVA-challenged WT mice (Supplementary Fig. S3c). We also performed immunohistochemistry for CCL11 (eotaxin-1) and CCL17 (Thymus and activation-regulated chemokine). CCL17 was expressed in AMs in OVA-challenged WT mice, but not in OVA challenged AQP3^−/−^ mice (Supplementary Fig. S4a). Although CCL17 was expressed in airway epithelial cells and type 2 alveolar cells in saline-exposed WT and AQP3^−/−^ mice, its expression was not enhanced in airway epithelial cells after OVA inhalation both in WT and AQP3^−/−^ mice. We obtained almost the same results for CCL11 (Supplementary Fig. S4b). These findings suggest that the production of several chemokines besides CCL24 from AMs was impaired in OVA-challenged AQP3^−/−^ mice, possibly due to their reduced activation.

### Regulation of chemokine production by AQP3 through the amount of cellular H_2_O_2_ in M2 polarized AMs

**Figure 6:**
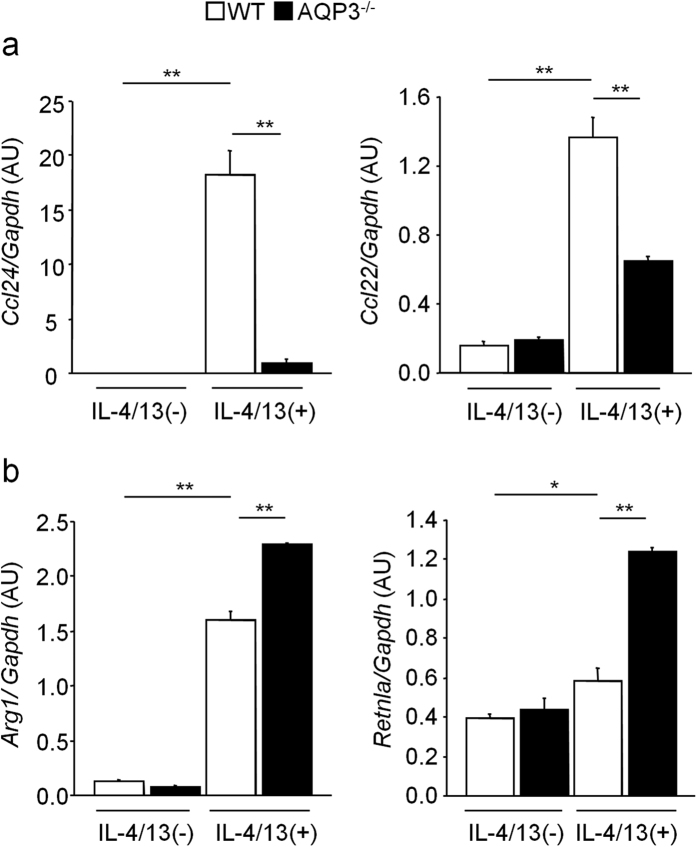
The expression of *Ccl24* and *Ccl22* mRNA is impaired in IL-4/13-stimulated AMs from AQP3^−/−^ mice. **(a)** mRNA expression levels of *Ccl24* and *Ccl22* in AMs *in vitro*. AMs isolated from naïve WT and AQP3^−/−^ mice were stimulated with IL-4/13 for 24 h (n = 3 for each group). **(b)** mRNA expression levels of *Arg1* and *Retnla* in AMs stimulated with/without IL-4/13 for 24 h *in vitro*. **P < 0.01, *P < 0.05.

To determine whether M2 polarization and chemokine production were due to different levels of stimulation of Th2 cytokines or the dysfunction of AMs, we isolated AMs from naïve WT and AQP3^−/−^ mice using FACS-Aria and stimulated them with the same levels of IL-4/13 for 24 h *in vitro*. As shown in Fig. 6a, *Ccl24* and *Ccl22* mRNA expressions in AQP3^−/−^ AMs were strongly suppressed after stimulation with IL-4/13 in comparison with WT AMs. However, contrarily, *Arg1* and *Retnla* mRNA expressions were rather increased in AQP3^−/−^ AMs compared to WT AMs (Fig. 6b), suggesting that chemokine production was seemingly regulated by AQP3 expression not through M2 polarization.

**Figure 7:**
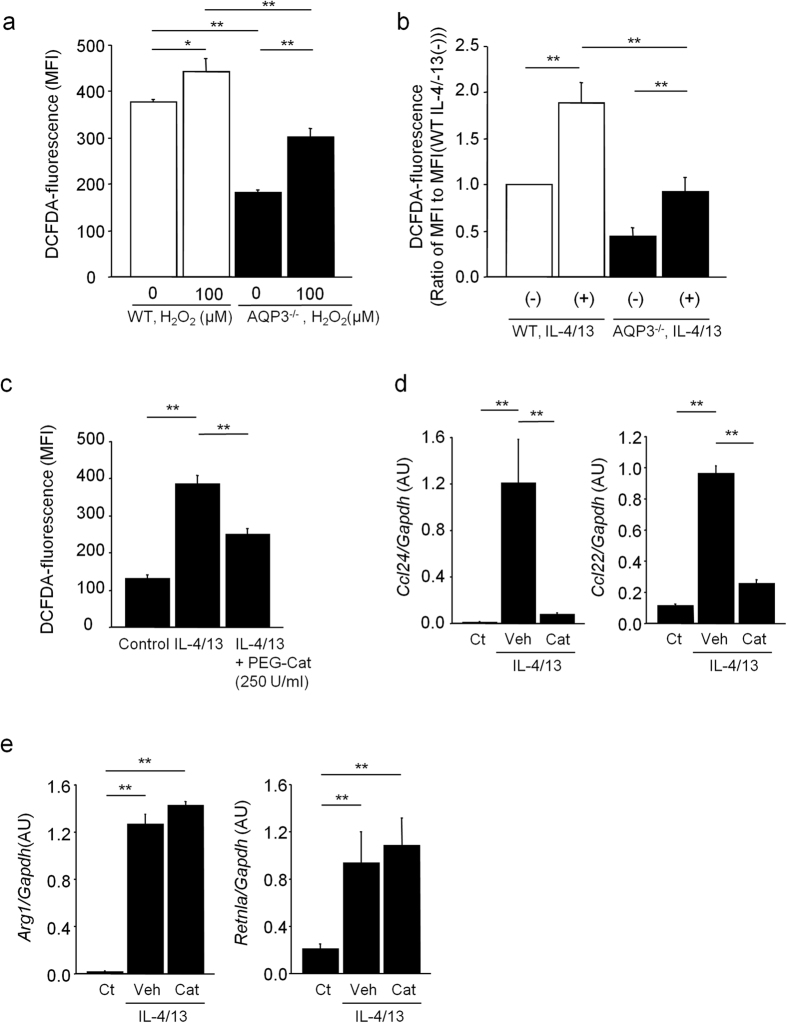
AQP3 regulates chemokine production from M2 polarized AMs through affecting the amount of cellular H_2_O_2_. **(a)** H_2_O_2_ permeability in naive AMs. Sorted AMs from naïve WT and AQP3^−/−^ mice were incubated with/without H_2_O_2_ (100 μM) for 15 s. Cellular H_2_O_2_ levels were determined using CM-H2DCFDA reagent by flow cytometry analysis. Mean fluorescence intensity (MFI) of CM-H2DCFDA is shown (n = 3 for each group). **(b)** Intracellular H_2_O_2_ levels in IL-4/13-stimulated AMs. Sorted AMs from naïve WT and AQP3^−/−^ mice were incubated with IL-4/13 for 24 h, and cellular H_2_O_2_ levels were determined (n = 4 for each group). As the numbers of AMs were small, we combined the results of 2 independent experiments. Ratios of MFI in each sample to MFI in naïve non-stimulated WT AMs are shown. **(c)** Cellular H_2_O_2_ levels in IL-4/13 stimulated AMs with/without PEG-catalase. Sorted AMs from naïve WT mice were incubated with 250 U/ml PEG-catalase for 1 h and followed with IL-4/13 for 24 h. Cellular H_2_O_2_ levels in AMs determined using CM-H2DCFDA reagent (n = 4–5 for each group). **(d**,**e)** mRNA expression levels of *Ccl24* and *Ccl22* (**d**), and *Arg1* and *Retnla* (**e**) in IL-4/13 stimulated AMs with/without PEG-catalase were measured by real-time RT-PCR (n = 4 for each group). **P < 0.01, *P < 0.05.

We anticipated that AQP3 would affect chemokine production from AMs by modulating the amount of cellular H_2_O_2_. Firstly, we measured intracellular H_2_O_2_ levels in AMs using 5-(and-6)-chloromethyl-2′,7′-dichlorodihydrofluorescein diacetate acetyl ester (CM-H2DCFDA) fluorescence. We found that cellular H_2_O_2_ levels were lower in naïve AQP3^−/−^ AMs than in naïve WT AMs without and with exogenous H_2_O_2_ supplementation for 15 s (Fig. 7a). Stimulation of AMs with IL-4/13 for 24 h increased cellular H_2_O_2_ levels in both AQP3^−/−^ and WT AMs, but its enhancement was more reduced in AQP3^−/−^ AMs (Fig. 7b). Thus, AQP3 was considered to be involved in regulating cellular H_2_O_2_ concentration in AMs under Th2 environment.

Next, we investigated whether the amount of cellular H_2_O_2_ would influence chemokine production in AMs without affecting M2 polarization. We stimulated naïve WT AMs with IL-4/13 with or without 1 h pretreatment with polyethylene glycol (PEG)-catalase, which decomposes hydrogen peroxide into water and oxygen. Pretreatment with PEG-catalase significantly suppressed an IL-4/13-induced increase in cellular H_2_O_2_ levels in WT AMs (Fig. 7c). Under the same condition, pretreatment with PEG-catalase significantly suppressed an IL-4/13-induced increase in *Ccl24* and *Ccl22* mRNA expressions, but not in *Arg1* and *Retnla* mRNA expressions in WT AMs (Fig. 7d,e). Collectively, our results suggest that AQP3 may regulate chemokine production from M2 polarized AMs under Th2 environment through modulating the amount of cellular H_2_O_2_.

## Discussion

Here we have revealed a critical role of AQP3 in OVA-induced asthma. Our results suggest that AQP3 potentiates asthma through mediating T cell trafficking and chemokine production from M2 polarized AMs by regulating the amount of cellular H_2_O_2_. Firstly, compared to WT mice, AQP3^−/−^ mice exhibited attenuated allergic airway inflammation as assessed by the number and composition of BALF cells, infiltration of inflammatory cells and mucus hypersecretion by histology, and Th2 cytokines in BALF. Secondly, adoptive transfer experiments demonstrated that the transfer of sensitized AQP3^−/−^ splenocytes had reduced airway eosinophilic inflammation due to impaired CD4^+^ T cell trafficking after OVA challenge. Thirdly, AQP3^−/−^ mice exhibited decreased levels of CCL24 and some chemokines in BALF, which was consistent with their lower gene expressions in AMs from AQP3^−/−^ mice than in those from WT mice. Fourthly, *in vitro* experiments revealed that chemokine expressions and intracellular H_2_O_2_ levels in Th2-cytokine-stimulated AMs were more greatly reduced in those from AQP3^−/−^ mice than in those from WT mice and were decreased after PEG-catalase without changes in M2 polarization markers.

Chemokines play an important role at multiple levels in asthma such as leukocyte recruitment and cellular activation^18^. Characteristically, the eotaxin family works as a potent chemoattractant for eosinophils^14^. CCL24 was reported to be mainly produced by AMs in murine experimental asthma^14^,^15^,^16^,^17^. We then first focused on CCL24 and showed that AQP3 mediates its production from AMs, probably contributing to eosinophilic inflammation. IL-4/IL-13 induces the differentiation of AMs towards M2 macrophages, and several chemokines were reported to be upregulated in murine M2 macrophages^3^. Moreover, in addition to CCL24, the levels of CCL17 and CCL22, which are chemoattractants for Th2 cells, in BALF were also reduced in OVA-challenged AQP3^−/−^ mice. Along with these three, some other chemokines produced by AMs were indicated to be more reduced in AQP3^−/−^ mice, including CCL2, CCL7, and CCL11, which were related to asthma at different levels^19^,^20^,^21^. Additionally, M2 macrophages are important in orchestration of tissue repair^3^. Microarray data and real-time RT-PCR analyses revealed that several genes related to tissue repair were reduced in AMs from AQP3^−/−^ mice. We also revealed that levels of pro-MMP-9 and TIMP-1 in BALF in OVA-challenged AQP3^−/−^ mice were significantly lower than those in WT mice. Previous studies showed that vascular endothelial growth factor, MMP-9, and TIMP-1 could be produced by AMs and contribute to airway remodeling^22^,^23^. Thus, AQP3 may mediate asthma partly through regulating the production of chemokines and tissue repair-related factors from AMs.

Several studies suggested that AM is one source of the increased ROS in asthmatics^24^. However, how H_2_O_2_ or ROS affects macrophage function is not well understood. Previous studies showed that H_2_O_2_ modulated the expression of some chemokines in rat AMs^25^,^26^ and increased mRNA of various chemokines in murine macrophages by activating extracellular signal-regulated kinase- and cyclic adenosine 5-monophosphate-dependent pathways^27^. Here we focused on the effect of the cellular H_2_O_2_ level in chemokine production from WT and AQP3^−/−^ AMs. Further, we found that, compared to WT AMs, the genes in the M2 marker were less upregulated in AQP3^−/−^ AMs in an *in vivo* asthma model, but, conversely, they were more upregulated by *in vitro* stimulation with IL-4/13. We interpret this inconsistency partly due to the lower level of Th2 inflammation in AQP3^−/−^ mice *in vivo*. We therefore considered that chemokine production from AMs was regulated by AQP3 expression possibly through facilitating H_2_O_2_ uptake independently of M2 polarization.

T cell trafficking is also crucial in the pathogenesis of asthma, as recruited T cells and their products then mediate airway eosinophilia, mucus hypersecretion, and AHR. While T cell proliferation and activation were similar between OVA-sensitized WT and AQP3^−/−^ mice during the sensitization phase, adoptive transfer experiments showed that, with OVA-sensitized AQP3^−/−^ donor splenocytes, total cells and eosinophils were reduced in BALF in recipient mice compared with respective results involving transferred WT donor splenocytes, suggesting the importance of AQP3 in airway inflammation through CD4^+^ T cell trafficking during the elicitation phase. Consistently, we reported that AQP3 facilitated chemokine-dependent T cell migration in contact hypersensitivity through mediating H_2_O_2_ transport, which was important for the activation of Cdc42 and F-actin dynamics^11^. Thus, AQP3 may be critical for T cell-mediated diseases^28^.

Epithelium is one of the main sources of chemokines and cytokines in asthma. Although we compared immunohistochemical staining for CCL11 and CCL17 between AQP3^−/−^ and WT mice, unlike in AMs, there was no difference in the expression of these chemokines in airway epithelium. We also measured the concentrations of IL-33, which is mainly released from epithelial cells, in BALF and found that the levels of IL-33 were comparable between AQP3^−/−^ and WT mice (Supplementary Fig. S5). Meanwhile, B cells synthesize IgE, which affects the pathogenesis of allergic diseases like asthma. Serum OVA-specific IgE levels after OVA challenge were less increased in AQP3^−/−^ mice than in WT mice. However, as B cells in BALF expressed *Aqp3* mRNA at a very low level by real-time RT-PCR, this would be due to reduced IL-4 and IL-13 release from Th2 cells. Thus, we consider that AQP3 may not directly contribute to OVA-induced asthma through its expression on epithelium or B cells although further investigation is needed.

Asthma is a complex disease and interactions between various cells can play important roles^29^. We interpreted the results with adoptive transfer experiments (Fig. 3c), where eosinophilic inflammation in AQP3^−/−^ recipient mice was more reduced than in comparative WT mice, by analyzing the functions of AMs. However, interactions of the inflammatory cells with epithelial cells, fibroblasts, endothelial cells, smooth muscle cells, etc. might also have affected our results in addition to the role of T cells and AMs.

Currently, asthma affects about 3 hundred million people worldwide. Its prevalence rate ranges from 1–18% in different countries and has been increasing in some countries^30^. Although inhaled corticosteroids are the mainstay for asthma treatment, 5–10% of patients have severe asthma, which does not respond to this treatment, and pulmonary macrophages may have roles in this resistance^31^. Some mediators discussed here such as neutrophil chemoattractants (CXCL1, CCL7, IL-17F) and those for tissue remodeling (MMP-9 and TIMP-1) were reported to be related to severe asthma^32^,^33^,^34^,^35^. High-dose or long-term corticosteroids use may have some systemic side effects. Therefore, AQP3 may be a novel and interesting therapeutic target for severe asthma.

In conclusion, our current findings provide evidence that AQP3 mediates not only T cell trafficking but chemokine production of M2 polarized AMs partly through regulating the amount of cellular H_2_O_2_, in the OVA-induced allergic asthma model. Recent studies reported confirmation of AQP3 inhibition by gold compounds^36^,^37^. Both T cells and macrophages also play a critical role in the pathogenesis of other allergic disorders such as allergic rhinitis and atopic dermatitis. Thus, AQP3 may represent a new therapeutic target for the treatment of allergic disorders overall, including asthma.

## Methods

### Mice

AQP3^−/−^ mice (C57BL/6 genetic background) were generated by targeted gene disruption^38^. We used C57BL/6 mice purchased from CLEA Japan (Tokyo, Japan) as WT control mice unless otherwise stated. All animal experimental protocols were approved by the Committee on Animal Research of Kyoto University, and performed in strict accordance with institutional guidelines.

### OVA-induced murine asthma

Mice were sensitized with intraperitoneal injection (i.p.) of 50 μg OVA (Sigma-Aldrich, St Louis, MO) and 1 mg alum (LSL, Tokyo, Japan) on day 0 and day 11, and then were challenged with aerosolized 1% OVA on days 21–27. Control mice received saline instead of OVA. Mice were sacrificed 24 h after the last challenge of OVA or saline (on day 28), then BALF, blood and lungs were harvested and analyzed (Supplementary Fig. S1a).

### Bronchoalveolar lavage

The trachea was cannulated and the lung was lavaged 3 times with 1 ml of PBS based on the modification of our previous procedure^39^. The BALF was centrifuged at 1300 rpm for 10 min at 4 °C, and the cell pellet and supernatant were saved separately. We suspended the pellet in 5% FBS-RPMI and used an aliquot for counting the number of nucleated cells using a hemocytometer or flow cytometry. We cytospinned an aliquot of cells and stained with Diff-quick (International Reagents, Kobe, Japan), a modified simple staining of May-Grünwald Giemsa. We then visually performed a differential leukocyte count among at least 200 cells under a microscope. We also examined an aliquot of cells using flow cytometry after staining with monoclonal antibodies against Thy1.2, B220, CD4, and CD8 (eBioscience, San Diego, CA).

### Lung histology and immunohistochemistry

24 h after the last challenge, lungs were distended by injection through the trachea of 10% formalin in PBS, and then were harvested. Murine lung tissues were embedded in paraffin and sliced into sections 4 μm thick, which were then stained with hematoxylin and eosin, periodic acid–Schiff, AQP3 antibody (Millipore, Billerica, MA), CCL11, and CCL17 (R&D Systems, Minneapolis, MN).

### Double staining of immunohistochemistry for AQP3 and F4/80

After deparaffinization, antigen retrieval treatment was performed by autoclaves in citrate buffer (pH 6.0). Then, the slides were incubated with a rabbit anti-mouse AQP3 antibody (dilution 1:500, Millipore), and thereafter with biotinylated anti-rabbit IgG antibody, followed by incubation with avidin-biotin-alkaline phosphatase complex (Vector Laboratories, Burlingame, CA). Color development was done with Fast red. Next, the slides were incubated with a rat anti-mouse F4/80 antibody (dilution 1:50, eBioscience), and thereafter with biotinylated anti-rat IgG antibody, followed by incubation with avidin-biotin-peroxidase complex (Vector Laboratories). Color development was done with 0.03% 3,3′-diaminobenzidine tetrahydrochloride (Dojindo Laboratories, Kumamoto, Japan). Finally, slides were counterstained with 0.1% Mayer’s hematoxylin.

### Measurement of airway resistance to methacholine

24 h after the last challenge, airway responses to aerosolized methacholine (Sigma-Aldrich) were measured using the Pulmos-I system (MIPS, Osaka, Japan). A mouse was placed in a double-flow plethysmographic chamber and exposed to saline and increasing concentrations of methacholine (1.56 to 25 mg/ml) for 1 min each concentration. We then measured sRaw in conscious mice according to a previously reported method^40^,^41^. We maintained the same air-conditioned environment controlled for temperature (22 °C) and humidity (50%) during the measurements to maintain reliability and reproducibility.

### Measurement of OVA-specific IgE in sera

Sera were collected before OVA challenge (on day 21) and after the last OVA challenge (on day 28), and the levels of OVA-specific IgE were determined using an ELISA kit (Shibayagi, Gunma, Japan).

### Cytokine and chemokine measurements

Cell-free BAL fluids or culture supernatants were analyzed for cytokine production. Concentrations of IL-4, IL-13, IL-17F, IFN-γ, and IL-33 were determined in a Bio-plex set (Bio-Rad, Richmond, CA). Concentrations of CCL24 were determined by ELISA (Raybio, Norcross, GA). Concentrations of CCL17 and CCL22 were determined using a MILLIPLEX MAP Mouse Cytokine/Chemokine Magnetic Bead panel (Millipore). Concentrations of pro-MMP-9 were determined using a MILLIPLEX MAP Mouse MMP Magnetic Bead panel (Millipore), and those of TIMP-1 were determined using a MILLIPLEX MAP Mouse Kidney Injury Magnetic Bead Panel 1 (Millipore). Concentrations of CCL2, CCL4, CCL7, CCL11, and CXCL1 were determined using ProcartaPlex Mouse Chemokine Panel 1 (9 plex) (eBioscience). Assays were used according to the respective manufacturer’s instructions.

### Quantitative real-time RT-PCR

RNA was extracted with Sepasol RNA I Super (Nacalai Tesque, Kyoto, Japan) from lung tissues and with the RNeasy Plus Micro Kit (Qiagen, Valencia, CA) from cells. cDNA was reverse transcribed from total RNA samples using a High-Capacity cDNA Reverse Transcription Kit (Applied Biosystems, Foster City, CA). Quantitative RT-PCR was performed using SYBR Premix Ex Taq (Takara Bio, Otsu, Japan) and the CFX96 real-time PCR system (Bio-Rad) according to the manufacturer’s instructions. Primers used in this study were shown in Supplementary Table S1.

### Cell isolation from BALF

CD4^+^ T cells, CD8^+^ T cells, and B cells were isolated by positive selection using magnetic cell sorting (Auto-MACS) after incubating BALF cells with magnetic beads labeled with CD4, CD8, and CD19 (Miltenyi Biotec, Bergisch Gladbach, Germany), respectively. The Auto-MACS system (Miltenyi Biotec) was used in accordance with the manufacturer’s instructions.

### OVA-specific cell responses

Based on our protocol for the OVA-induced asthma model shown in Supplementary Fig. S1a, mice were sensitized with 50 μg OVA and 1 mg alum on days 0 and 11, and on day 21 splenocytes and mesenteric lymph nodes cells were collected and cultured in the presence of 100 μg/ml OVA in complete RPMI (RPMI 1640 supplemented with 10% heat-inactivated FBS, 50 μM 2-mercaptoethanol (Wako, Osaka, Japan), 25 mM HEPES, 100 μM nonessential amino acids, 10 μM sodium pyruvate (Invitrogen, Carlsbad, CA), and 1% penicillin/streptomycin (Gibco Invitrogen, Carlsbad, CA)) for 72 h. The OVA-specific cell proliferative responses were determined by incorporation of 0.5 μCi/ml [^3^H]-thymidine for 24 h.

### Adoptive transfer experiments

On day 21 after WT and AQP3^−/−^ mice were sensitized intraperitoneally with OVA and alum, splenocytes were collected and stimulated with 200 μg/ml OVA in complete RPMI for an additional 3 days (Supplementary Fig. S1b). After stimulation with OVA, cell suspensions were intravenously (1 × 10^7^ cells/head) injected to naïve WT and AQP3^−/−^ mice. One day later, the recipient mice were challenged with aerosolized 1% OVA for 7 days. 24 h after the last challenge, BALF was harvested and analyzed. Additionally, to track the transferred cells, OVA-sensitized cells derived from WT and AQP3^−/−^ mice were stained with CMFDA (Invitrogen) for 20 min, washed, and intravenously (2 × 10^7^ cells/head) injected to naïve WT mice (Supplementary Fig. S1c). After the cell transfer, mice were challenged with aerosolized 1% OVA for 3 days. Six h after the last challenge, thoracic lymph nodes and lungs were harvested, and CD4^+^ and CMFDA^+^ cells were analyzed by flow cytometry.

### AM isolation from lungs

To obtain lung cell suspensions, we removed and minced lungs from mice. The minced materials were incubated in complete RPMI and collagenase A (Roche, Indianapolis, IN) for 1 h at 37 °C and digests were filtered through a nylon mesh (40 μm). Dissociated cells were centrifuged at 1300 rpm for 10 min at 4 °C, and the cell pellet was incubated for 5 min in room air with ACK lysing buffer (Life Technologies, Grand Island, NY) to lyse RBCs. The single cell suspension was labeled with FITC-conjugated CD11c (eBioscience) and PE-conjugated F4/80 (Biolegend). AMs were sorted based on their expression of CD11c and F4/80^42^ by FACS Aria (BD Biosciences, San Jose, CA).

### DNA microarray analysis

RNA was extracted from AMs of WT and AQP3^−/−^ mice before and after the OVA challenge (days 21 and 28). We prepared 4–6 mice for each group and pooled their samples. Total RNA was converted to cDNA and was amplified using the Ovation Pico WTA System V2 (NuGen, San Carlos, CA). The cDNA was then labeled with Cyanine-3 using a SureTag Complete DNA Labeling Kit (Agilent, Santa Clara, CA). Labeled DNA was hybridized to SurePrint G3 Mouse GE 8 × 60 K Microarray (Agilent). Agilent Feature Extraction software was used for normalization, filtering, and initial quality control assessment of the genechip data. We selected 8,458 genes that differed in signal intensity both by more than 4-fold (fold change) and by a difference of more than 100 in comparison of minimum from maximum signals, and performed hierarchical cluster analysis on the selected genes (Supplementary Fig. S3a). Furthermore, we performed hierarchical cluster analysis again on 2,591 genes in clusters 9 and 11. Raw data were deposited in the Gene Expression Omnibus database: accession no. GSE68420.

### AM culture

AMs were isolated as described above and were cultured in complete RPMI. Cells were stimulated with 10 or 50 ng/ml IL-4 and IL-13 for 24 h to induce M2 macrophages. For some experiments, cells were pretreated with 250 units/ml PEG-catalase (Sigma-Aldrich) for 1 h before the culture.

### H_2_O_2_ permeability assay

Cellular H_2_O_2_ was assayed using the CM-H2DCFDA reagent (Invitrogen) according to the manufacturer’s instructions.

### Statistical analysis

We made comparisons among data with either the Student’s t test or analysis of variance. We considered *P* values of less than 0.05 to be statistically significant.

## Additional Information

**How to cite this article**: Ikezoe, K. *et al*. Aquaporin-3 potentiates allergic airway inflammation in ovalbumin-induced murine asthma. *Sci. Rep.* **6**, 25781; doi: 10.1038/srep25781 (2016).

## Supplementary Material

Supplementary Information

## Figures and Tables

**Table 1:** Selective list of chemokine-related genes in alveolar macrophages from OVA-challenged WT (WT OVA (+)) versus non-OVA-challenged WT (WT OVA (−)) or OVA-challenged AQP3^−/−^ mice (AQP3^−/−^OVA (+)).

Genbank Access	Description	Symbol	Level of mRNA (ratio)	
			WT OVA (+) /WT OVA (−)	WT OVA (+) /AQP3^−/−^OVA (+)
NM_013652	chemokine (C-C motif) ligand 4	*Ccl4*	3.4	2.7
NM_021443	chemokine (C-C motif) ligand 8	*Ccl8*	44.0	4.6
NM_011338	chemokine (C-C motif) ligand 9	*Ccl9*	5.1	1.8
NM_011330	chemokine (C-C motif) ligand 11	*Ccl11*	7.3	3.1
NM_011331	chemokine (C-C motif) ligand 12	*Ccl12*	24.3	5.5
NM_011332	chemokine (C-C motif) ligand 17	*Ccl17*	12.9	1.8
NM_008176	chemokine (C-X-C motif) ligand 1	*Cxcl1*	2.9	5.7
NM_009141	chemokine (C-X-C motif) ligand 5	*Cxcl5*	7.3	2.1
AK156907	chemokine (C-X-C motif) ligand 10	*Cxcl10*	12.3	2.7
NM_023158	chemokine (C-X-C motif) ligand 16	*Cxcl16*	10.3	3.6

WT, wild-type; OVA, ovalbumin.

Ratio of respective mRNA is shown. Genes listed in Fig. 5a were not shown here.

## References

[b1] Medoff B. D., Thomas S. Y. & Luster A. D. T cell trafficking in allergic asthma: The ins and outs. Annu Rev Immunol 26, 205–232 (2008). 18304002 10.1146/annurev.immunol.26.021607.090312

[b2] Holgate S. T. & Polosa R. Treatment strategies for allergy and asthma. Nat Rev Immunol 8, 218–230 (2008). 18274559 10.1038/nri2262

[b3] Martinez F. O., Helming L. & Gordon S. Alternative activation of macrophages: An immunologic functional perspective. Ann Rev Immunol 27, 451–483 (2009). 19105661 10.1146/annurev.immunol.021908.132532

[b4] Lee I. T. & Yang C. M. Role of NADPH oxidase/ROS in pro-inflammatory mediators-induced airway and pulmonary diseases. Biochem Pharmacol 84, 581–590 (2012). 10.1016/j.bcp.2012.05.005 22587816

[b5] Kirkham P. & Rahman I. Oxidative stress in asthma and COPD: Antioxidants as a therapeutic strategy. Pharmacol Ther 111, 476–494 (2006). 10.1016/j.pharmthera.2005.10.015 16458359

[b6] Verkman A. S., Anderson M. O. & Papadopoulos M. C. Aquaporins: Important but elusive drug targets. Nat Rev Drug Discov 13, 259–277 (2014). 10.1038/nrd4226 PMC4067137 24625825

[b7] Rojek A., Praetorius J., Frøkiaer J., Nielsen S. & Fenton R. A. A current view of the mammalian aquaglyceroporins. Annu Rev Physiol 70, 301–327 (2008). 17961083 10.1146/annurev.physiol.70.113006.100452

[b8] Hara M., Ma T. & Verkman A. S. Selectively reduced glycerol in skin of aquaporin-3-deficient mice may account for impaired skin hydration, elasticity, and barrier recovery. J Biol Chem 277, 46616–46621 (2002). 12270942 10.1074/jbc.M209003200

[b9] Hara M. & Verkman A. S. Glycerol replacement corrects defective skin hydration, elasticity, and barrier function in aquaporin-3-deficient mice. Proc Natl Acad Sci USA 100, 7360–7365 (2003). 12771381 10.1073/pnas.1230416100 PMC165880

[b10] Miller E. W., Dickinson B. C. & Chang C. J. Aquaporin-3 mediates hydrogen peroxide uptake to regulate downstream intracellular signaling. Proc Natl Acad Sci USA 107, 15681–15686 (2010). 20724658 10.1073/pnas.1005776107 PMC2936599

[b11] Hara-Chikuma M. . Chemokine-dependent T cell migration requires aquaporin-3-mediated hydrogen peroxide uptake. J Exp Med 209, 1743–1752 (2012). 10.1084/jem.20112398 PMC3457725 22927550

[b12] Krane C. M. . Altered regulation of aquaporin gene expression in allergen and IL-13-induced mouse models of asthma. Cytokine 46, 111–118 (2009). 10.1016/j.cyto.2008.12.018 PMC2703176 19237298

[b13] Dong C. . Anti-asthmatic agents alleviate pulmonary edema by upregulating AQP1 and AQP5 expression in the lungs of mice with OVA-induced asthma. Respir Physiol Neurobiol 181, 21–28 (2012). 10.1016/j.resp.2011.12.008 22226856

[b14] Pope S. M., Zimmermann N., Stringer K. F., Karow M. L. & Rothenberg M. E. The eotaxin chemokines and CCR3 are fundamental regulators of allergen-induced pulmonary eosinophilia. J Immunol 175, 5341–5350 (2005). 10.4049/jimmunol.175.8.5341 16210640

[b15] Pope S. M. . Identification of a cooperative mechanism involving interleukin-13 and eotaxin-2 in experimental allergic lung inflammation. J Biol Chem 280, 13952–13961 (2005). 10.1074/jbc.M406037200 15647285

[b16] Kurowska-Stolarska M. . IL-33 amplifies the polarization of alternatively activated macrophages that contribute to airway inflammation. J Immunol 183, 6469–6477 (2009). 10.4049/jimmunol.0901575 19841166

[b17] Crapster-Pregont M., Yeo J., Sanchez R. L. & Kuperman D. A. Dendritic cells and alveolar macrophages mediate IL-13-induced airway inflammation and chemokine production. J Allergy Clin Immunol 129, 1621–1627 (2012). 10.1016/j.jaci.2012.01.052 PMC3583235 22365581

[b18] Zimmermann N., Hershey G. K., Foster P. S. & Rothenberg M. E. Chemokines in asthma: Cooperative interaction between chemokines and IL-13. J Allergy Clin Immunol 111, 227–243 (2003). 10.1067/mai.2003.139 12589338

[b19] Schneider D. . Macrophage/epithelial cell CCL2 contributes to rhinovirus-induced hyperresponsiveness and inflammation in a mouse model of allergic airways disease. Am J Physiol Lung Cell Mol Physiol 304, L162–L169 (2013). 10.1152/ajplung.00182.2012 PMC3567365 23204071

[b20] Rojas-Ramos E. . Role of the chemokines RANTES, monocyte chemotactic proteins-3 and -4, and eotaxins-1 and -2 in childhood asthma. Eur Respir J 22, 310–316 (2003). 10.1183/09031936.03.00084802 12952266

[b21] Pilette C., Francis J. N., Till S. J. & Durham S. R. CCR4 ligands are up-regulated in the airways of atopic asthmatics after segmental allergen challenge. Eur Respir J 23, 876–884 (2004). 10.1183/09031936.04.00102504 15219001

[b22] Lee C. G. . Vascular endothelial growth factor (VEGF) induces remodeling and enhances TH2-mediated sensitization and inflammation in the lung. Nat Med 10, 1095–1103 (2004). 10.1038/nm1105 PMC3434232 15378055

[b23] Kelly E. A. & Jarjour N. N. Role of matrix metalloproteinases in asthma. Curr Opin Pulm Med 9, 28–33 (2003). 10.1097/00063198-200301000-00005 12476081

[b24] Bowler R. P. Oxidative stress in the pathogenesis of asthma. Curr Allergy Asthma Rep 4, 116–122 (2004). 10.1007/s11882-004-0056-7 14769260

[b25] Shi M. M., Godleski J. J. & Paulauskis J. D. Regulation of macrophage inflammatory protein-1α mRNA by oxidative stress. J Biol Chem 271, 5878–5883 (1996). 10.1074/jbc.271.10.5878 8621460

[b26] Shi M. M., Chong I. W., Godleski J. J. & Paulauskis J. D. Regulation of macrophage inflammatory protein-2 gene expression by oxidative stress in rat alveolar macrophages. Immunology 97, 309–315 (1999). 10.1046/j.1365-2567.1999.00798.x PMC2326837 10447747

[b27] Jaramillo M. & Olivier M. Hydrogen peroxide induces murine macrophage chemokine gene transcription via extracellular signal-regulated kinase- and cyclic adenosine 5′-monophosphate (cAMP)-dependent pathways: Involvement of NF-κB, activator protein 1, and cAMP response element binding protein. J Immunol 169, 7026–7038 (2002). 10.4049/jimmunol.169.12.7026 12471138

[b28] Hara-Chikuma M. . Aquaporin-3-mediated hydrogen peroxide transport is required for NF-kappaB signalling in keratinocytes and development of psoriasis. Nat Commun 6, 7454 (2015). 10.1038/ncomms8454 PMC5628617 26100668

[b29] Siddiqui S., Hollins F., Saha S. & Brightling C. E. Inflammatory cell microlocalisation and airway dysfunction: cause and effect? Eur Respir J 30, 1043–1056 (2007). 10.1183/09031936.00162506 18055703

[b30] Bateman E. D. . Global strategy for asthma management and prevention: GINA executive summary. Eur Respir J 31, 143–178 (2008). 10.1183/09031936.00138707 18166595

[b31] Yang M., Kumar R. K., Hansbro P. M. & Foster P. S. Emerging roles of pulmonary macrophages in driving the development of severe asthma. J Leukoc Biol 91, 557–569 (2012). 10.1189/jlb.0711357 22293472

[b32] Michalec L. . CCL7 and CXCL10 orchestrate oxidative stress-induced neutrophilic lung inflammation. J Immunol 168, 846–852 (2002). 10.4049/jimmunol.168.2.846 11777981

[b33] Al-Ramli W. . TH17-associated cytokines (IL-17A and IL-17F) in severe asthma. J Allergy Clin Immunol 123, 1185–1187 (2009). 10.1016/j.jaci.2009.02.024 19361847

[b34] Fitzpatrick A. M., Higgins M., Holguin F., Brown L. A. S. & Teague W. G. The molecular phenotype of severe asthma in children. J Allergy Clin Immunol 125, 851–857, e818 (2010). 10.1016/j.jaci.2010.01.048 PMC2851636 20371397

[b35] Ko F. W. S. . A comparison of airway and serum matrix metalloproteinase-9 activity among normal subjects, asthmatic patients, and patients with asthmatic mucus hypersecretion. Chest 127, 1919–1927 (2005). 10.1378/chest.127.6.1919 15947303

[b36] Martins A. P. . Aquaporin inhibition by gold(III) compounds: New insights. ChemMedChem 8, 1086–1092 (2013). 10.1002/cmdc.201300107 23653381

[b37] Serna A. . Functional inhibition of aquaporin-3 with a gold-based compound induces blockage of cell proliferation. J Cellular Physiol 229, 1787–1801 (2014). 10.1002/jcp.24632 24676973

[b38] Ma T. . Nephrogenic diabetes insipidus in mice lacking aquaporin-3 water channels. Proc Natl Acad Sci USA 97, 4386–4391 (2000). 10.1073/pnas.080499597 PMC18251 10737773

[b39] Oga T. . Prostaglandin F_2α_ receptor signaling facilitates bleomycin-induced pulmonary fibrosis independently of transforming growth factor-Β. Nat Med 15, 1426–1430 (2009). 10.1038/nm.2066 19966781

[b40] Pennock B. E., Cox C. P., Rogers R. M., Cain W. A. & Wells J. H. A noninvasive technique for measurement of changes in specific airway resistance. J Appl Physiol Respir Environ Exerc Physiol 46, 399–406 (1979). 10.1152/jappl.1979.46.2.399 422457

[b41] Flandre T. D., Leroy P. L. & Desmecht D. J. M. Effect of somatic growth, strain, and sex on double-chamber plethysmographic respiratory function values in healthy mice. J Appl Physiol 94, 1129–1136 (2003). 10.1152/japplphysiol.00561.2002 12571140

[b42] Bedoret D. . Lung interstitial macrophages alter dendritic cell functions to prevent airway allergy in mice. J Clin Invest 119, 3723–3738 (2009). 10.1172/JCI39717 PMC2786798 19907079

