# Supplementary material for: Aquaporin-3 potentiates allergic airway inflammation in ovalbumin-induced murine asthma
Source: Sci Rep. 2016 May 11;6:25781. doi: 10.1038/srep25781 (PMC4863152; doi:10.1038/srep25781)
Supplement: Supplementary Information [file srep25781-s1.pdf]

Supplementary Information

Aquaporin-3 potentiates allergic airway inflammation in ovalbumin-induced murine asthma

Kohei Ikezoe[1], Toru Oga*[2], Tetsuya Honda[3, 4], Mariko Hara-Chikuma[3, 4], Xiaojun Ma[5], Tatsuaki Tsuruyama[6], Kazuko Uno[7], Jun-ichi Fuchikami[8], Kiminobu Tanizawa[2], Tomohiro Handa[1], Yoshio Taguchi[9], Alan S. Verkman[10], Shuh Narumiya[4], Michiaki Mishima[1], and Kazuo Chin[2]

[1]Department of Respiratory Medicine, Kyoto University Graduate School of Medicine, Sakyo-ku, Kyoto 606-8507, Japan

[2]Department of Respiratory Care and Sleep Control Medicine, Kyoto University Graduate School of Medicine, Sakyo-ku, Kyoto 606-8507, Japan

[3]Department of Dermatology, Kyoto University Graduate School of Medicine, Sakyo-ku, Kyoto 606-8507, Japan

[4]Center for Innovation in Immunoregulative Technology and Therapeutics (AK project), Kyoto University Graduate School of Medicine, Sakyo-ku, Kyoto 606-8501, Japan

[5]Core Research for Evolutional Science and Technology (CREST) Laboratory, Medical Innovation Center, Kyoto University Graduate School of Medicine, Sakyo-ku, Kyoto 606-8501, Japan

[6]Center for anatomical, forensic and pathology research, Kyoto University Hospital, Sakyo-ku, Kyoto 606-8501, Japan

[7]Louis Pasteur Center for Medical Research, Sakyo-ku, Kyoto 606-8225, Japan

[8]Bioresearch Center, CMIC Pharma Science Co., Ltd., Hokuto-shi, Yamanashi 408-0044, Japan

[9]Department of Respiratory Medicine, Tenri Hospital, Tenri-shi, Nara 632-8552, Japan

[10]Departments of Medicine and Physiology, University of California, San Francisco, CA 94143, USA

Supplementary Figure S1

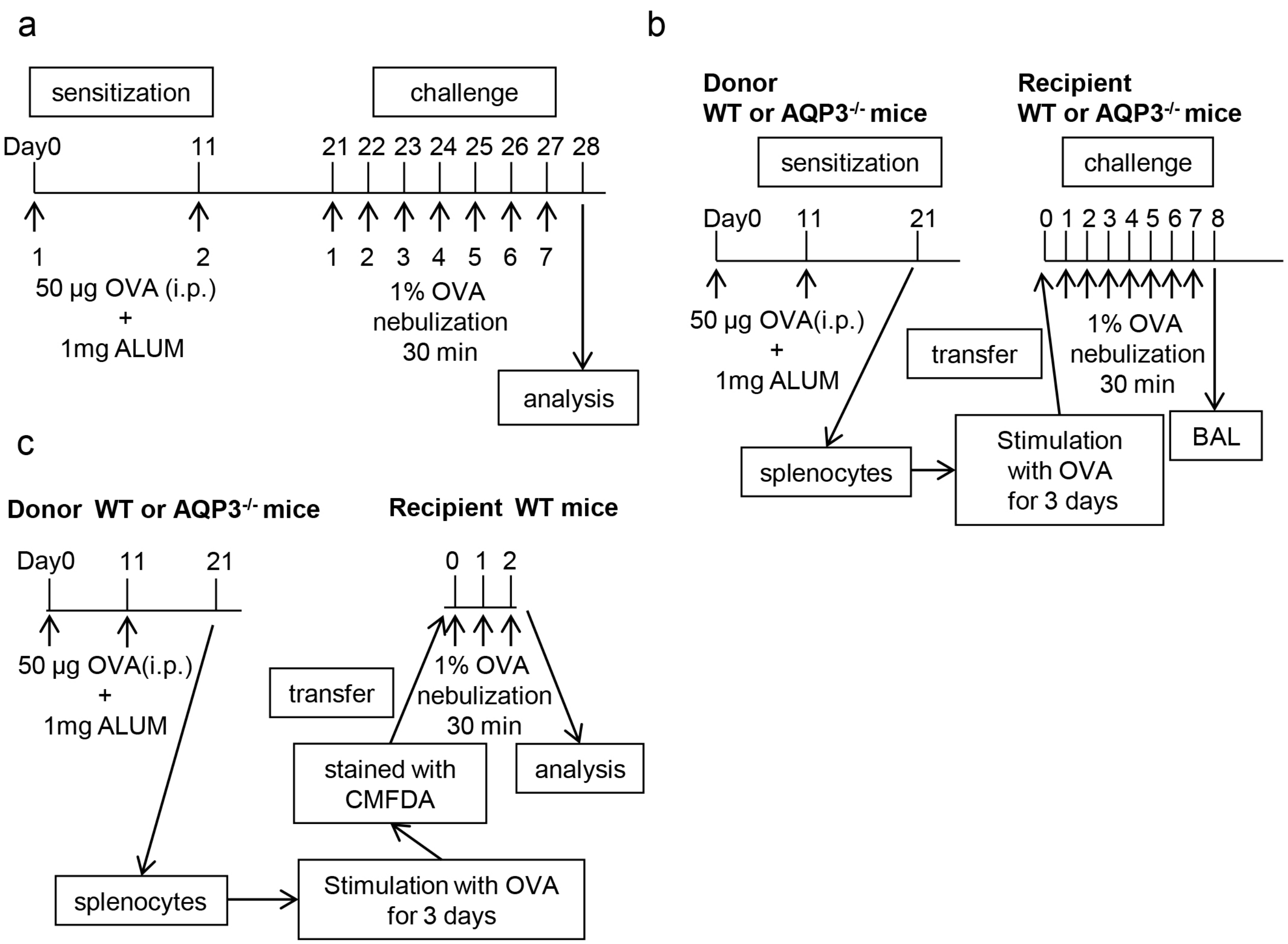

Supplementary Figure S1. Diagrammatic representation of the protocols for OVA-induced asthma model and adoptive transfer models. (**a**) Protocol for OVA sensitization and challenge. (**b**) Protocol for transfer of the OVA-sensitized splenocytes. (**c**) Protocol to track transferred splenocytes.

Supplementary Figure S2

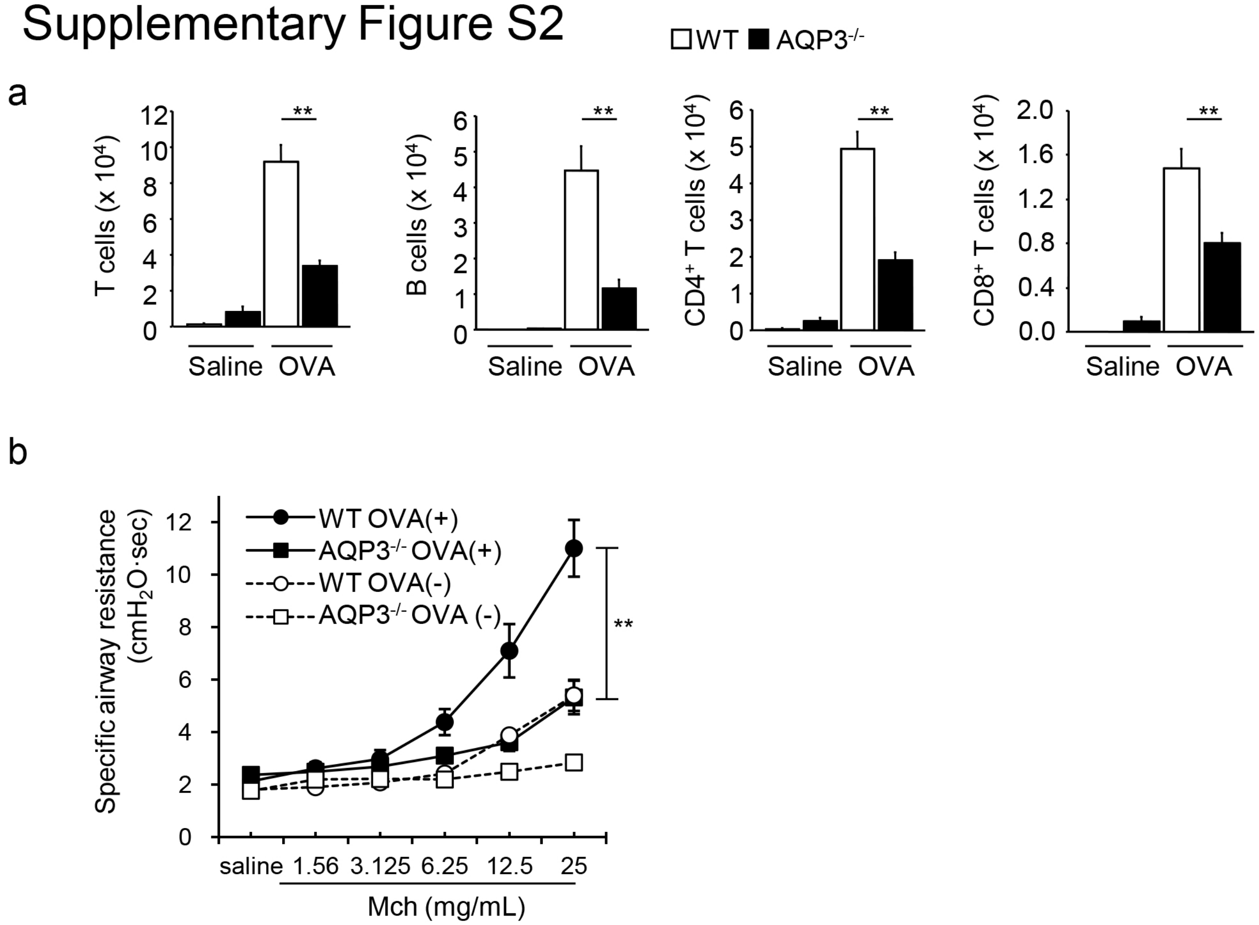

Supplementary Figure S2. (a) Lymphocyte analysis in BALF by flow cytometry in OVA-induced models. BALF was obtained from WT and AQP3$^{-/-}$ mice on day 28, and cell population analysis in BALF by flow cytometry is shown (n = 5 for each control group; n = 8 for each OVA-challenged group). **(b) Airway responsiveness to methacholine.** Dose-response curves of methacholine (Mch)-induced airway resistance in non-OVA-challenged (n = 3-4 for each group) and OVA-challenged (n = 6 for each group) WT or AQP3$^{-/-}$ mice. **, $P < 0.01$.

Supplementary Figure S3

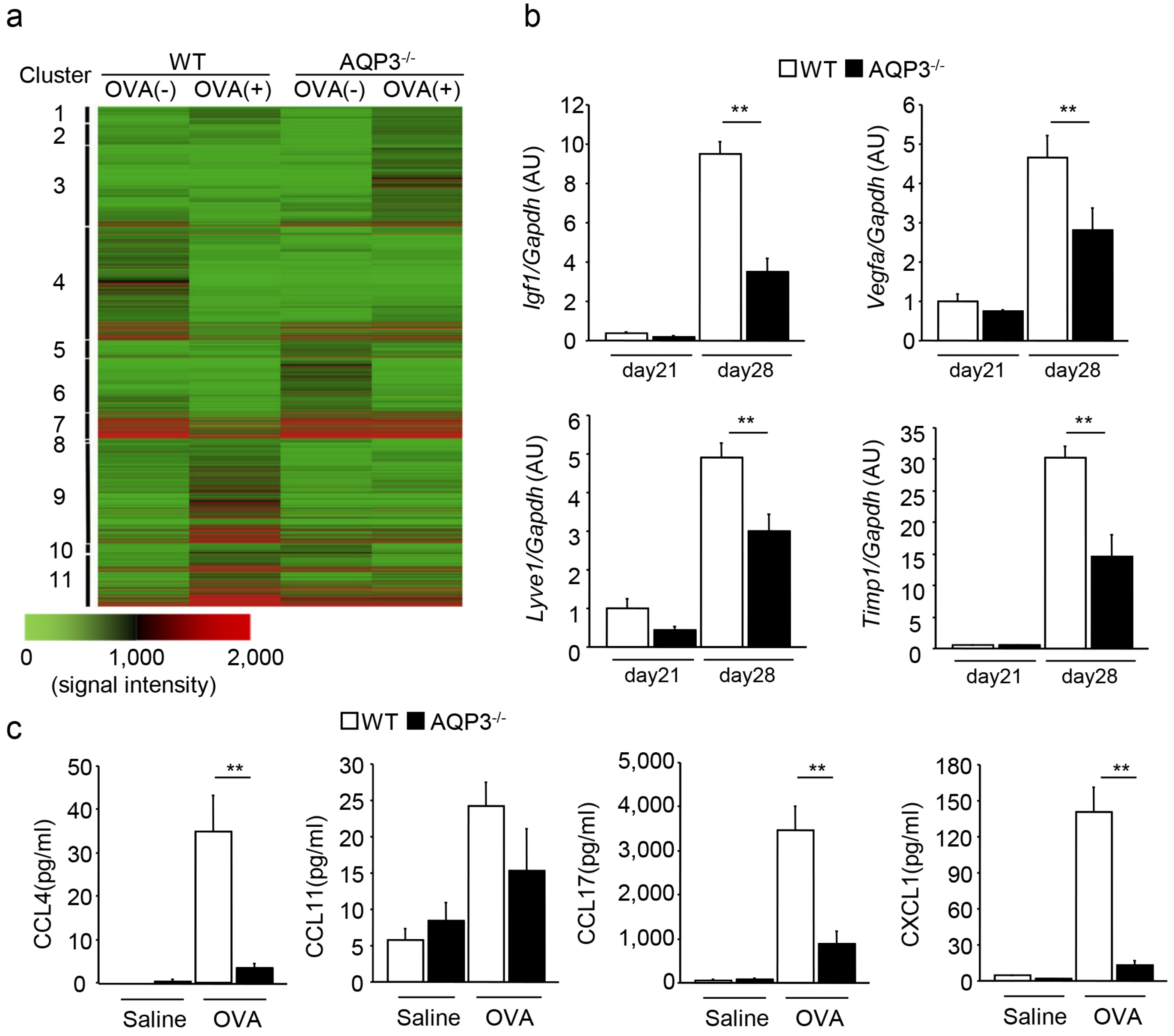

Supplementary Figure S3. (a) Cluster analysis. RNA was prepared from AMs of OVA-challenged (on day 28) or non-OVA-challenged (on day 21) WT and AQP3$^{-/-}$ mice. 4-6 mice for each group were prepared and samples were pooled. After normalization and filtering, we selected 8,458 genes, and performed hierarchical cluster analysis on the selected genes. Expression of genes belonging to each cluster is shown with graded color from green to red. **(b) mRNA expression levels of the genes related to tissue remodeling in AMs.** *Igf1, Vegfa, Lyve1, and Timp1* mRNA expression levels in AMs from OVA-challenged WT and AQP3$^{-/-}$ mice (n = 4 on day 21; n = 6 on day 28). **(c) Chemokine levels of BALF in WT and AQP3$^{-/-}$ mice.** Levels of CCL4, CCL11, CCL17, and CXCL1 in BALF after the last OVA challenge (day 28) (n = 5 for each control group; n = 8 for each OVA-challenged group). **, $P < 0.01$.

Supplementary Figure S4

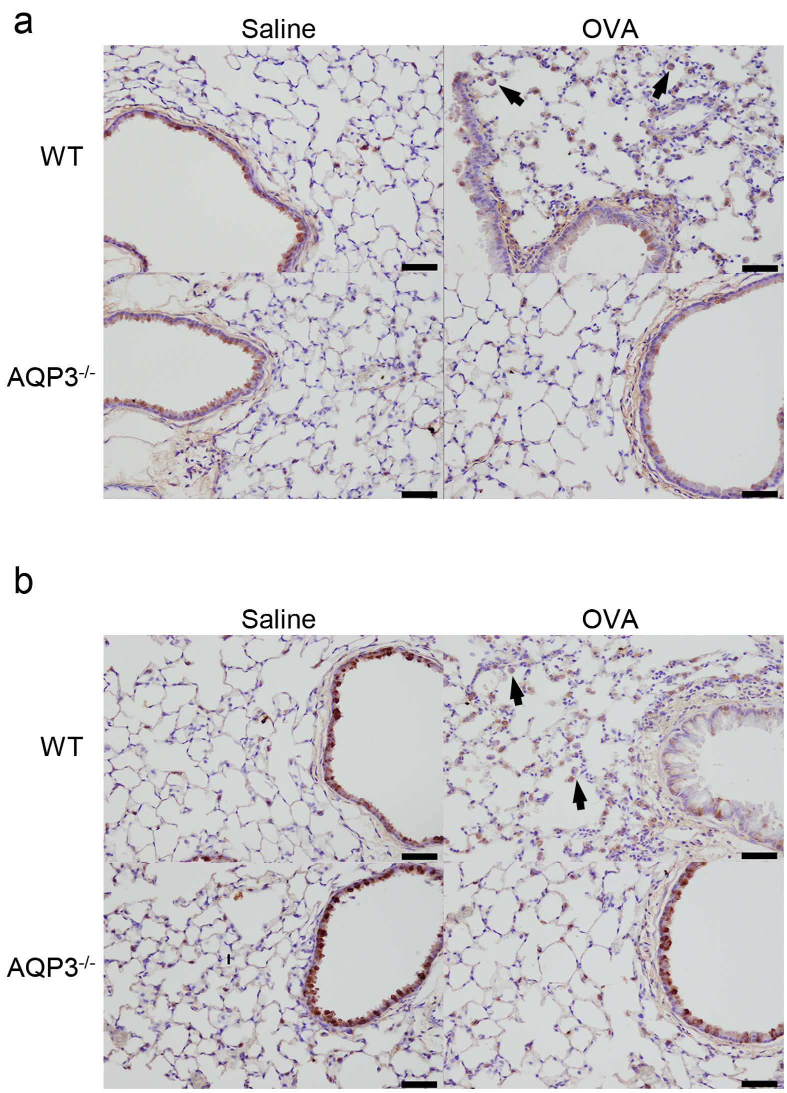

Supplementary Figure S4. (a, b) Immunohistochemical staining for CCL17 and CCL11 in WT and AQP3$^{-/-}$ mice. Expressions of CCL17 (a) and CCL11 (b) in lung tissues of control and OVA-challenged WT and AQP3$^{-/-}$ mice were evaluated by immunohistochemical staining. Arrows indicate AMs. Bars, 20 μm.

Supplementary Figure S5

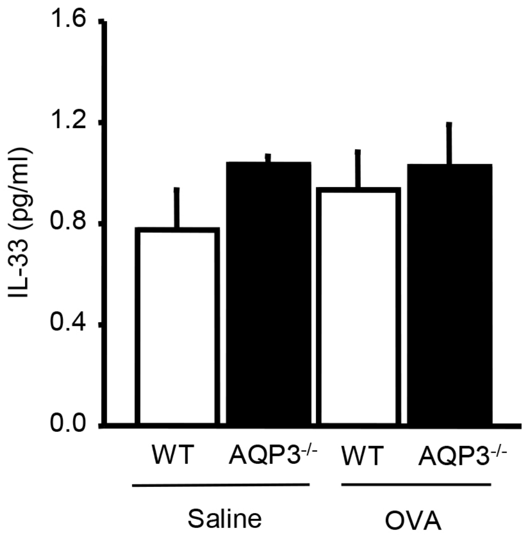

Supplementary Figure S5. The levels of IL-33 in BALF from OVA-induced asthma model mice.

BALF were collected on day 28 (n = 5 for each control group; n =8 for each OVA-challenged group).

Supplementary Table S1. Primers used in this study.

Gene	Direction	Sequence
Aqp3	Forward	5'-GCT TTT GGC TTC GCT GTC AC-3'
	Reverse	5'-TAG ATG GGC AGC TTG ATC CAG-3'
Arg1	Forward	5'-CTC CAA GCC AAA GTC CTT AGA G-3'
	Reverse	5'-AGG AGC TGT CAT TAG GGA CAT C-3'
Ccl2	Forward	5'-CAG GTC CCT GTC ATG CTT C-3'
	Reverse	5'-TGA GTG GGG CGT TAA CTG C-3'
Ccl22	Forward	5'-CAT CAT GGC TAC CCT GGG TGT CCC-3
	Reverse	5'-CCT CCT CCC TAG GAC AGT TTA TGG A-3'
Ccl24	Forward	5'-CTG CCT TCT GGG ATG TCT GG-3'
	Reverse	5'-TCA GAT GGT GGT GTG CTT GC-3'
Ccl7	Forward	5'-ACC AAC CTA GGA GCC AAG AAG CAA-3'
	Reverse	5'-AAG ACC ATT CCT TAG GCG TGA CCA-3'
Gapdh	Forward	5'-GGG CCA TCC ACA GTC TTC TG-3'
	Reverse	5'-TGA TGG GTG TGA ACC ACG AG-3'
Igf1	Forward	5'-CAA CTC CCA GCT GTG CAA TT-3'
	Reverse	5'-GCC GAG GTG AAC ACA AAA CT-3'
Lyve1	Forward	5'-AGG AGC CCT CTC CTT ACT GC-3'
	Reverse	5'-ACC TGG AAG CCT GTC TCT GA-3'
Retnla	Forward	5'-GTG TCA AGA CTA TGA ACA GAT G-3'
	Reverse	5'-TGG TCC AGT CAA CGA GTA-3'
Timp1	Forward	5'-CCA CCC ACA GAC AGC CTT CT-3'
	Reverse	5'-CTG GTA TAA GGT GGT CTC GTT GAT T-3'
Vegfa	Forward	5'-ACT GGA CCC TGG CTT TAC TG-3'
	Reverse	5'-TCT GCT CTC CTT CTG TCG TG-3'
